# Genus *Paracoccidioides*: Species Recognition and Biogeographic Aspects

**DOI:** 10.1371/journal.pone.0037694

**Published:** 2012-05-30

**Authors:** Raquel Cordeiro Theodoro, Marcus de Melo Teixeira, Maria Sueli Soares Felipe, Karina dos Santos Paduan, Paulo Martins Ribolla, Gioconda San-Blas, Eduardo Bagagli

**Affiliations:** 1 Universidade Estadual Paulista, Campus de Botucatu-UNESP, São Paulo, Brazil; 2 Universidade de Brasília – UNB, Distrito Federal, Brazil; 3 Venezuelan Institute for Scientific Research, Center of Microbiology and Cell Biology, Caracas, Venezuela; New York State Health Department and University at Albany, United States of America

## Abstract

**Background:**

Paracoccidioidomycosis is a systemic mycosis caused by *Paracoccidioides brasiliensis* (species S1, PS2, PS3), and *Paracoccidioides lutzii*. This work aimed to differentiate species within the genus *Paracoccidioides*, without applying multilocus sequencing, as well as to obtain knowledge of the possible speciation processes.

**Methodology/Principal Findings:**

Single nucleotide polymorphism analysis on *GP43, ARF* and PRP8 intein genes successfully distinguished isolates into four different species. Morphological evaluation indicated that elongated conidia were observed exclusively in *P. lutzii* isolates, while all other species (S1, PS2 and PS3) were indistinguishable.

To evaluate the biogeographic events that led to the current geographic distribution of *Paracoccidioides* species and their sister species, Nested Clade and Likelihood Analysis of Geographic Range Evolution (LAGRANGE) analyses were applied. The radiation of *Paracoccidioides* started in northwest South America, around 11–32 million years ago, as calculated on the basis of *ARF* substitution rate, in the BEAST program. Vicariance was responsible for the divergence among S1, PS2 and *P. lutzii* and a recent dispersal generated the PS3 species, restricted to Colombia. Taking into account the ancestral areas revealed by the LAGRANGE analysis and the major geographic distribution of *L. loboi* in the Amazon basin, a region strongly affected by the Andes uplift and marine incursions in the Cenozoic era, we also speculate about the effect of these geological events on the vicariance between *Paracoccidioides* and *L. loboi*.

**Conclusions/Significance:**

The use of at least 3 SNPs, but not morphological criteria, as markers allows us to distinguish among the four cryptic species of the genus *Paracoccidioides*. The work also presents a biogeographic study speculating on how these species might have diverged in South America, thus contributing to elucidating evolutionary aspects of the genus *Paracoccidioides*.

## Introduction

The genus *Paracoccidioides* encompasses thermo-dimorphic fungal pathogens from the family Ajellomycetaceae, order Onygenales [1]; its species grow as yeast cells at 37°C or in mammal tissues, and as mycelia, producing the infective asexual spores or conidia, at 25°C or in soil [2], [3]. All members of this family, which also includes the pathogenic species *Blastomyces dermatitidis* (teleomorph *Ajellomyces dermatitidis*), *Histoplasma capsulatum* (teleomorph *Ajellomyces capsulatus*), *Emmonsia parva* and *E. crescens* (teleomorph *Ajellomyces crescens*), might have evolved in close association with vertebrate hosts, presenting a saprobic phase in soil and/or feces and a parasitic phase in host tissue [1], [4], [5]. In recent studies, the pathogenic fungus *Lacazia loboi* was also included in this group, as a *Paracoccidioides* sister species [6], [7]. This fungus, incapable of growing in culture media, is known to cause a subcutaneous mycosis in dolphins and humans, especially those from the Amazon basin [8].

Until 2006, the genus *Paracoccidioides* was believed to include only one species, *Paracoccidioides brasiliensis*, as the etiologic agent of paracoccidioidomycosis (PCM), one of the most frequent systemic mycoses in Latin America [2]. This pathogen has been repeatedly recovered from human clinical samples and tissues of some armadillo species, such as the nine-banded armadillo, *Dasypus novemcinctus* [9] and occasionally from *Cabassus centralis* [10]. It was also detected in and isolated from dogs [11], [12]. Molecular polymorphism analysis suggested that the same “ecopathogenotypes” can infect humans and other mammals [13]. It was only through Multi Locus Sequence Typing (MLST) analysis that the genetic variability, formerly known as merely intraspecific and geographic polymorphism, revealed the existence of four cryptic species: S1, PS2 and PS3, from the *P. brasiliensis* complex [14], [15] and *Paracoccidioides lutzii* [7], [16] (originally called Pb01-like). A formal description of the latter has been submitted for publication by Teixeira et al. S1 is the most widely distributed species, occurring in Brazil, Argentina, Paraguay, Uruguay, Peru and Venezuela; PS2 occurs in Venezuela and Brazil, in sympatry with S1, while PS3 is restricted to Colombia. Recombination events in *P. brasiliensis* species S1, PS2 and *P. lutzii* were detected by partition homogeneity test and split decomposition method while the *P. brasiliensis* species PS3 was considered to be clonal [14], [7]. *P. lutzii*, the most recently discovered species, is the most divergent and occurs predominantly, though not exclusively, in the western-central region of Brazil [7].

The species concept applied to the four cryptic species of *P. brasiliensis* was the phylogenetic one, which detects genetic divergence among populations through the Multi Locus Sequence Typing, by concordance of gene genealogies [17], [18].

Although there is no clear agreement on whether S1, PS2 and PS3 are geographical variants of one single species [19] or distinctly separated species [14], [15], these clades and *P. lutzii* are reproductively isolated in nature, as revealed by Split Decomposition Analysis [14], [7]. Since reproductive or genetic isolation is the first step in species divergence [18], this might eventually lead to morphological and physiological differences, with important consequences for the diagnosis and treatment of PCM. For instance, Batista Jr. et al. [20] demonstrated that the *Paracoccidioides* standard *gp43* reference antigen for PCM, when tested against sera from patients living in geographically distant areas, produces a high frequency of false negative results in immunodiffusion tests, possibly due to the assays having been performed in different *Paracoccidioides* species. In another study, Carvalho et al. [21] observed that PS2 isolates showed low virulence when inoculated in B10.A mice by the intraperitoneal, intratracheal and intravenous routes, which could be related to the down-regulation of Pb*GP43* observed in Pb03, a PS2 isolate (and not in Pb18 or Pb339, S1 isolates) as a result of heat shock at 42°C and temperature shift to prompt a mycelium-to-yeast transition. Another study, published before the discovery of cryptic species in the *Paracoccidioides* genus, which used RAPD markers, had already separated a group of isolates from western-central Brazil (denominated cluster II and now known as *P. lutzii*) from the rest of *P. brasiliensis* isolates. These isolates were more susceptible in vitro to trimethoprim-sulfamethoxazole and also produced a better response in vivo than isolates from other regions [22].

This work aimed to evaluate morphological and molecular markers for fast species recognition in the genus *Paracoccidioides*. Morphological analysis included yeast cell morphometry and conidial production in the mycelial phase of isolates from each species. For molecular analysis, SNPs from the genes *GP43* (43 KDa immunodominant glycoprotein), *ARF* (ADP-ribosylation factor) [23], [24], [25], [14] and PRP8 intein (intervening parasitic genetic element from the PRP8 gene) [26] were analyzed. Additionally, phylogenetic data and geographic locations of every isolate were associated by Nested Clade (NCA) and Likelihood Analysis of Geographic Range Evolution analysis, in an attempt to resolve the ancestral areas and the main biogeographic events that might have taken place during the radiation of this genus in South America and its divergence from its sister species, *L. loboi*.

## Materials and Methods

### Fungal isolates and growth conditions

Sixty-three isolates were used for mycological and molecular studies (Table 1). Of these, 26 S1, 9 PS2, 2 PS3 and 5 *P. lutzii* isolates were used for morphological studies. For species identification with SNPs as molecular markers, 34 S1 and 8 PS2 isolates were used, with 4 of each being identified by MLST [14] (positive controls). All PS3 and *P. lutzii* isolates (3 and 5, respectively) were used as positive controls (Table 1).

**Table 1:** Isolates from *Paracoccidioides* genus used in this work for morphological and molecular analysis and their species identification.

Species	Isolate	Origin	Host	Isolation source	Method for species identification*		
					MLST	SNaPshot	Real Time PCR-TaqMan
S1	T1F1 #	Pratânea, SP, Brazil	*D. novemcinctus*	Liver	x		
	T3B6 #	Pratânea, SP, Brazil	*D. novemcinctus*	Spleen	x	x	x
	T4B17 #	Manduri, SP, Brazil	*D. novemcinctus*	Spleen	x		x
	T5LN1 #	Botucatu, SP, Brazil	*D. novemcinctus*	Mesenteric lymph node	x		
	T7F6 #	Manduri, SP, Brazil	*D. novemcinctus*	Liver	x		
	T8LN2 #	Botucatu, SP, Brazil	*D. novemcinctus*	Mesenteric lymph node	x		
	T9B1 #	Botucatu, SP, Brazil	*D. novemcinctus*	Spleen	x		
	T13LN2 #	Manduri, SP, Brazil	*D. novemcinctus*	Mesenteric lymph node	x		
	T15LN1 #	Manduri, SP, Brazil	*D. novemcinctus*	Mesenteric lymph node	x		
	Bt60 #	Botucatu, SP, Brazil	Human	NK	x		
	Pb18 #	NK	Human	NK	x		
	Pb339 #	Brazil	Human	NK	x		
	S1 #	São Manoel, SP, Brazil	*D. novemcinctus*	Spleen		x	x
	ILSL57 #	Bauru, SP, Brazil	*D. novemcinctus*	Spleen		x	x
	Pb-baby	Ribeirão Preto, SP, Brazil	Human	NK		x	x
	Bt85 #	Botucatu, SP, Brazil	Human	NK		x	x
	Pb265 #	Botucatu, SP, Brazil	Human	NK		x	x
	D01 #	Avaré, SP, Brazil	Human	Cutaneous lesion		x	x
	D02 #	Laranjal Paulista, SP, Brazil	Human	Cutaneous lesion		x	x
	D03 #	Piracicaba, SP, Brazil	Human	Cutaneous lesion		x	x
	D04 #	Pereiras, SP, Brazil	Human	Cutaneous lesion		x	x
	D05 #	Botucatu, SP, Brazil	Human	Cutaneous lesion		x	x
	D06 #	Elias Fausto, SP, Brazil	Human	Cutaneous lesion		x	x
	D07 #	Assis, SP, Brazil	Human	Cutaneous lesion		x	x
	D09 #	Limeira, SP, Brazil	Human	Cutaneous lesion		x	x
	D10 #	Riversul, SP, Brazil	Human	Cutaneous lesion		x	x
	D11 #	Iperó, SP, Brazil	Human	Cutaneous lesion		x	x
	EPM04	Pará, Brazil	*D. novemcinctus*	NK	x	x	x
	EPM46	Argentina	Human	NK		x	x
	EPM48	Argentina	Human	NK		x	x
	EPM85	Peru	Human	Lips	x	x	x
	EPM75	Venezuela (1994)	Human	mouth		x	x
	EPM101	Ibiá, MG, Brazil	*D. novemcinctus*	NK		x	x
	Pb21	Bauru, SP, Brazil, 1964	Human	NK		x	x
	Pb534	Barquissimeto, Venezuela	Human	NK		x	x
	Pb113	Manaus-AM, Brazil, 1971	Human	NK		x	x
	Pb157	Uruguay	Human	NK		x	x
	Pb94	Belo Horizonte, MG, 1971	Human	NK		x	x
	Pb135	PA, Brazil	NK	NK		x	x
	Pb25	MG, Brazil	Human 19 years	NK		x	x
	Pb09	Uruguay, 1925	Human	NK		x	x
	Pb728	Venezuela, 1982	Human	NK		x	x
	Pb698	Venezuela, 1982	Human	NK		x	x
	TCC	Cerqueira César, SP, Brazil	*D. novemcinctus*	Mesenteric lymph node		x	x
PS2	T10B1 #	Botucatu, SP, Brazil	*D. novemcinctus*	Spleen	x	x	x
	Pb927 *** #		Antarctica/Uruguay	Pinguin	Feces	x	x
	Bt84 #	Botucatu, SP, Brazil	Human	NK	x	x	x
	Pb262 #	Uberlândia, MG, Brazil	-	Dog food	x	x	x
	Pb02 #	Caracas, Venezuela	Human	NK	x		
	Pb03 #	São Paulo, SP, Brazil	Human	NK	x		
	Pb04 #	São Paulo, SP, Brazil	Human	NK	x		x
	Pbdog #	Curitiba, PR, Brazil	Canis familiaris	Popliteal lymph node		x	x
	1430 #	Santa Maria, RS, Brazil	Human	Tongue		x	
	Pb106	Piraju, SP, Brazil, 1970	Human, 17 years	NK		x	x
	Pb22	Bauru, SP, Brazil, 1964	Human	NK		x	x
PS3	EPM83 #	Colombia (CIB)	Human	NK	x	x	x
	EPM77 #	Colombia (CIB)	NK	NK	x		x
	60855	Colombia (CIB)	NK	NK	x	x	
*P. lutzii*	Pb01 #	Goiania, GO, Brazil	Human	NK	x	x	x
	Pb66 #	Goiania, GO, Brazil	Human	NK	x	x	x
	8334 #	Goiania, GO, Brazil	Human	NK	x	x	x
	JAL** #	Cuiabá, MT, Brazil	Human	NK	x	x	x
	EE #	Cuiabá, MT, Brazil	Human	NK	x	x	x

#: isolates used for morphological analysis.

* The MLST was previously carried out by Matute et al. (2006) and Teixeira et al. (2009). The SNPs were detected in this work.

** Isolate whose morphometry of yeast cell was not studied due to the slow mycelia-yeast transition.

*** Isolate identified as S1 by Matute et al. (2006), but clustered with PS2 isolates according to the SNP analysis and sequencing of the intein PRP8 (Theodoro et al., 2008). NK: Not Known.

The yeast phase was maintained in GPYA (2% glucose, 1% peptone, 0.5% yeast extract and 1% agar) at 36°C, and the mycelial phase in PDA (Potato dextrose agar – Oxoid Lyd., Basingstoke, UK), at 25°C.

### Yeast cell morphometry

Morphometric studies of the yeast phase were carried out to test the hypothesis that *P. lutzii* isolates have yeast cells significantly larger than those of any *P. brasiliensis* isolate [7]. Yeast cells on the 7th day of growth were stained with lactophenol cotton blue for microscopic analysis in five fields, chosen at random. About 30 yeast cells were measured in each microscopic field. Two diameter measures of each cell were registered onto an Excel spreadsheet to determine the area, according to the following formula: (longest diameter×0.5)×(shortest diameter×0.5)×3.1416. The relationship between area and species was evaluated by using the statistical software R. Microscopic observation and measures were carried out in a Photomicroscope (Olympus, BX60); using the software Leica Qwin V3.

### Conidial production

The production and shape of conidia seemed to be an interesting morphological characteristic for differentiating species in the genus *Paracoccidioides*, given that Teixeira et al. (2009) reported longer conidia in *P. lutzii*. To induce conidial production, isolates were kept at 25°C for 30–40 days as giant colonies in Petri dishes, both on PDA and Soil Extract Agar (SEA), prepared according to Kwon-Chung [27]. Production of conidia was evaluated by using adhesive tape (Durex, 3 M), which was softly touched onto the surface of the colonies, previously killed by formaldehyde vapor for 48 h, and also by the slide culture technique, using both culture media, followed by microscopic observation as described above.

### Single nucleotide polymorphism (SNP) analysis

This was performed on isolates listed in the last two columns of Table 1. DNA was extracted according to McCullough et al. [28], quantified in Nanovue (GE Healthcare) and diluted in ultra-pure water to 15 ng/µL. SNP analysis was carried out by SNaPshot® Multiplex System, and/or Real Time PCR with TaqMan® Probes (Applied Biosystems). For the SNaPshot technique, the *ARF* gene and the *GP43* exon 2 were amplified by PCR. Reactions were carried out in 25 µl of reaction mixture containing 10 ng genomic DNA, 1× PCR buffer (50 mMKCl, 10 mMTrisHCl and 1.5 mM MgCl2), 0.2 mMdNTP, 10 pmoles of each primer and 1 unit of Taq polymerase (GE Healthcare). Amplifications were performed in a thermocycler (Eppendorf) in the following sequence: 94°C for 4 min, 40 cycles of 94°C for 1 min, 55°C for 1 min, 72°C for 2 min and a final cycle at 72°C for 5 min. Primer sequences and amplicon lengths are listed in Table S1.

*ARF* and *GP43* sequences, previously obtained by (Matute et al. [14] for isolates belonging to S1, PS2 and PS3 species, and by Teixeira et al. [7] for *P. lutzii* isolates, were aligned by using Clustal W algorithm, implemented in Mega v3.0 software [29]. Species-specific SNPs were selected for species recognition of 34 unidentified *Paracoccidioides* isolates. For SNaPshot, forward primers (SNP-primers) were designed on flanking SNPs regions. Cytosines were added to their 5′ end so that the amplified fragments could differ in length and position in the polyacrylamide gel. Primer sequences, number of added cytosines, SNP positions and expected SNP for each species are listed in Table 2.

**Table 2:** SNPs evaluated by SNaPshot and Real Time PCR techniques; primers and probes used and expected SNP for each species.

Gene	SNP position*	SNaPshot Primer	Real Time PCR – Taqman				SNPs/species			
			VIC probe	FAM probe	Forward primer (5′-3′)	Reverse Primer (5′-3′)	S1	PS2	PS3	*P. lutzii*
*GP43*-exon2	344	4(C)GTATCTAAATGGGCGTGGCA	-	-	-	-	**T**	**T**	**T**	**G**
	494	-	CATCCAGCCGCGACCT	CATCCAGCCCCGACCT	GCGCTTGTGGTGCGA	CTCCGTCTTCCATGTCCAGAA	**G**	**G**	**C**	**G**
*ARF* **	298	21(C)CATCCAATTTACCGTGTGGGA	-	-	-	-	**C**	**T**	**C**	**C**
		-	CCACCCACGTCCCACA	CCACCCACATCCCACA	GCAGGATTTAACGTCGAGACTGT	GCTGTCAACCACGAAAATGATACCT	**G**	**A**	**G**	**G**
PRP8 intein	1146	-	TCCTCCTCACCAGGACA	CTCCTCGCCAGGACA	GCGAACTGGTTGTCGAACGTA	GGCCAACGATATCTTCAAGGATTTG	**A**	**A**	**A**	**G**

* According to the sequences deposited by Matute et al. (2006) and Theodoro et al. (2008) in GenBank.

** The same SNP was, in this case, studied by using the SNaPshot and Real Time PCR (TaqMan) methodologies, in the two DNA strands.

SNaPshot reactions were carried out as follows: 6 µL of ultra pure water, 1 µL of ½ TERM buffer (200 mMTrisHCl, 5 mM MgCl2, pH 9.0), 1 µL of SNaPmix (Applied Biosystems), 1 µL of SNP-primer (1 pmol/µL) and 1 µL of the PCR product, previously purified with ExoSAP-IT (GE Healthcare) according to the supplier's instructions. Reactions were performed in a Whatman Biometra® (T Gradient) thermocycler using the following conditions: 40 cycles 95°C for 10 sec, 50°C for 5 sec and 60°C for 30 sec.

Since free ddNTPs can co-migrate with the fragment of interest, the 5′phosphoryl groups were removed by enzymatic treatment with SAP (shrimp alkaline phosphatase, GE Healthcare), according to the supplier's instructions. Reaction samples were diluted 1∶1 with Loading Buffer [45% HI DI formamide (v/v) (Applied Biosystems), 2.26 µM EDTA pH 8.0 and 2.26% Blue Dextran (w/v)]. Samples were heated at 95°C for 3 min and placed on ice. One microliter of each sample was loaded onto 12% polyacrylamide gel and submitted to electrophoresis in an ABI PRISM 377 DNA sequencer (Applied Biosystems). The SNPs were analyzed according to their colors.

For Real Time PCR, TaqMan probes, as well as forward and reverse primers for *GP43*, *ARF* and PRP8 intein, were designed (Table 2). Polymorphic sites of the PRP8 intein had been previously evaluated [24]. For each reaction, 1.5 µL ultra-pure water, 5 µL Master Mix (Fermentas), 0.5 µL 20× primers and probe mixture (Applied Biosystems) and 3 µL DNA diluted to 15 ng/µL, were used. Thermal cycling conditions were 95°C for 10 min, followed by 40 cycles at 92°C for 15 sec and 60°C 1 min, in the Step One Plus ™ equipment (Applied Biosystems). To determine SNPs, endpoint fluorescence was measured by means of the Step One Plus software (Applied Biosystems).

### Biogeographic analysis

Nested Clade Analysis (NCA) in ANeCA and NETWORK software packages: To study the association between phylogenetic data and geographic location of 84 isolates (Table S2; sequences obtained previously by Matute et al. [14] and Teixeira et al. [7]), the software ANeCA [30]–[32] was used. Nucleotide sequences from *ARF* (407 nt), *GP43* (427 nt from exon 2) and *TUB* (α-tubulin) (243 nt) were used in separate analyses. A haplotype genealogy, a population distribution profile for each haplotype and a nested design (hierarchical clustered haplotypes known as “clades”) were constructed by using the TCS and nesting algorithms. The geographic information (latitude, longitude and the approx. 200 Km2 range of isolate occurrence) was used as an input file for running the geographic association test in the program GeoDis. Finally, the automated inference key was run. A haplotype net was also constructed by using the software NETWORK v.4.6.0.0 (downloaded from http://www.fluxus-engineering.com), according to the Median-Joining algorithm, which allows multi-state data [33]on the haplotypes to be represented in a pie chart, whose slice sizes are proportional to the number of isolates, with different colors, according to the geographic origin of isolates in each haplotype.

### Estimation of divergence time and Likelihood Analysis of Geographic Range Evolution

**Figure 1:**
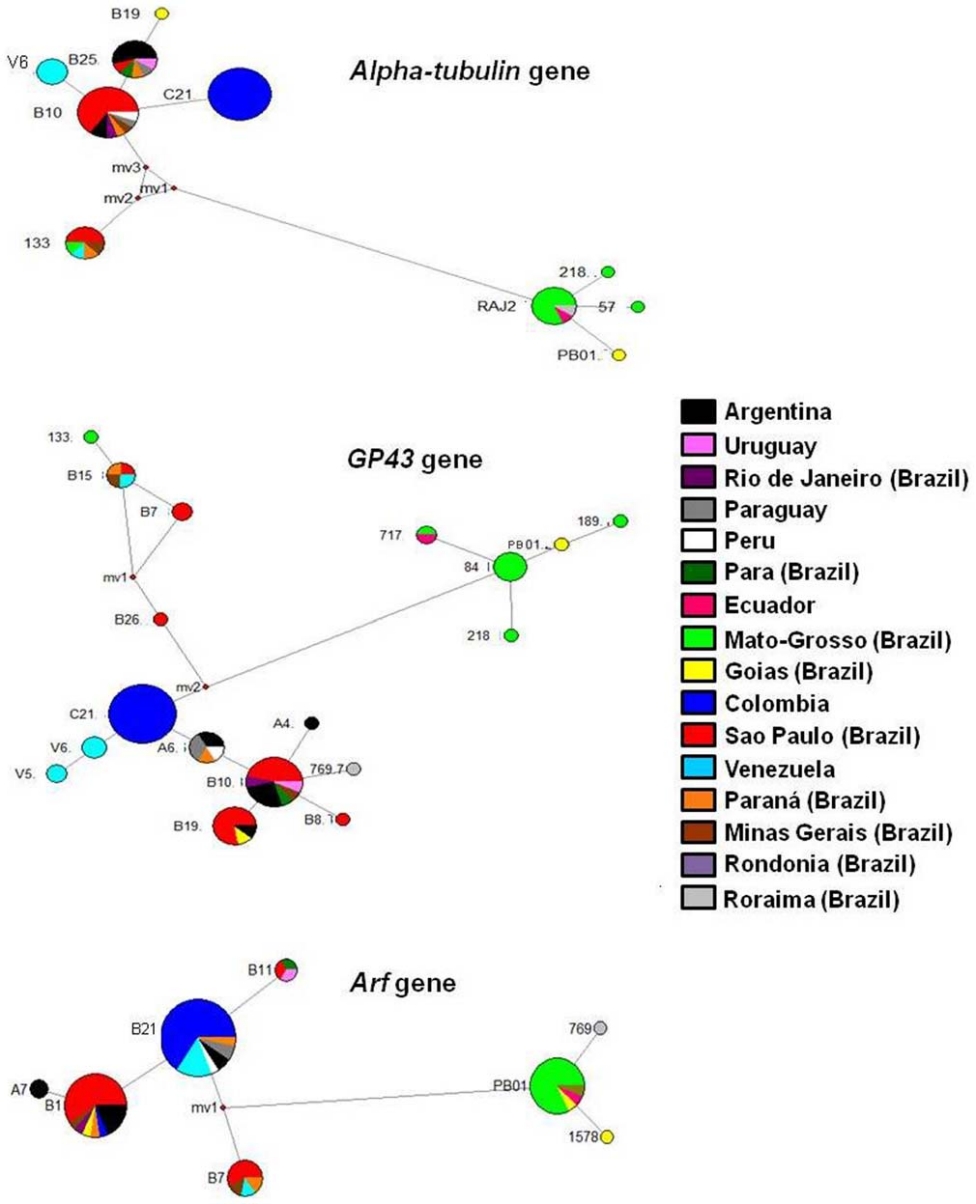
Haplotype nets generated by ANeCA and NETWORK programs, for *Alpha-tubulin, GP43* and *ARF* genes. Each haplotype is indicated by a circle, whose colored slices represent geographic distribution, according to the legend on the right side. mv: median vector = nodes and links which connect the nodes. It is a hypothesized ancestral sequence required to connect existing sequences within the network with Maximum Parsimony.

*ARF* nucleotide sequences from each *Paracoccidioides* phylogenetic species (8 haplotype sequences, the same haplotypes obtained by NCA and NETWORK analyses, shown in Figure 1 and Table 3) plus *L. Loboi* (GenBank access number EU096466) were used for this analysis because its DNA substitution rate had been previously determined [34]. We estimated the radiation time for *Paracoccidioides* species and the divergence time between this genus and *L. loboi*, using the BEAST software v1.4.8 (downloaded from http://beast.bio.ed.ac.uk) [35]. Using BEAUTI (a program distributed with BEAST), an XML input file was generated to run in BEAST, adopting HKY as the evolution model, with an estimated base frequency but without gamma site heterogeneity or invariant sites. The DNA substitution rate per site per year used was 0.51×10–9 to 1.11×10–9, as previously calculated by Kasuga et al. [34] for the *ARF* gene. Since our data consist of a species-level phylogeny, the Yule tree prior, which specifies a constant rate of species divergence, was selected. To create the tree, 10 million generations were run and 1 tree was collected per 1000 generations. Stable likelihood plateaus from independent runs were recognized using the graphing function in Tracer (a program also distributed with BEAST), where the first one million generations were discarded as burn-in. The mean likelihood was −720.5 and the ESS (Effective Sample Size), an estimate of the number of independent samples of the posterior distribution, was higher than the acceptable level (200) according to the BEAST manual. In the program TreeAnotator v1.4.8, also distributed with BEAST, the first 1000 trees were ignored and the tree with maximum credibility (Highest Log Clade Credibility: 1.725) was found. The tree, with node ages, was visualized in the program FigTree v.1.3.1, downloaded from http://tree.bio.ed.ac.uk/software/figtree/.

**Table 3:** Isolates belonging to haplotypes obtained for Alpha-tubulin, *GP43* and *ARF* gene alignments.

Gene	Haplotype	Isolates
*Alpha-Tubulin*	B10	B1,B2,B3,B4,B5,B6,B8,B9,B10,B12,B14,B16,B17,B20,B21,B22,A1,A6,P1,PE1
	V6	V1,V3,V4,V5,V6
	B25	B11,B24,B25,A2,A3,A4,A5,A7,A8,P2,U1
	B19	B19
	133	133,B7,B15,B23,B26,V2,PBDOG,B13
	RAJ2	RAJ2,717,84,189,61,206,694,397,351,769,7455
	218	218
	57	57
	PB01	PB01
	C21	C1,C2,C3,C4,C5,C6,C7,C8,C9,C10,C11,C12,C13,C14,C15,C16,C17,C18,C19,C20,C21
*GP43*	B10	B10,B9,B6,B22,B25,B17,A3,A7,A5,A2,B16,B11,B24,B12,U1
	B19	B19,B1,B5,B4,B3,B2,B14,B20,A2
	A4	A4
	769	769
	B8	B8
	A6	A6,A1,PE1,B21,P1,P2
	C21	C1,C2,C3,C4,C5,C6,C7,C8,C9,C10,C11,C12,C13,C14,C15,C16,C17,C18,C19,C20,C21
	V6	V6,V4,V3
	V5	V5,V1
	B15	B15,V2,PBDOG,B13
	B7	B7,B23
	133	133
	B26	B26
	717	717,7455
	84	84,206,57,397,RAJ2
	PB01	PB01
	189	189
	218	218
*ARF*	A7	A7,A8
	B1	B1,B2,B3,B4,B5,B6,B8,B9,B10,B12,B14,B16,B17, B19,B20,B22,B24,C10,A2,A3,A4,A5
	B21	B21,C1,C2,C3,C4,C5,C6,C7,C8,C9,C11,C12,C13,C14,C15,C16,C17,C18,C19,C20,C21,V1,V3,V4,V5,V6,A1,A6, P1,P2,PE1
	B11	B25,B11,U1
	B7	B7,B15,B13,B23,B26,PBDOG,V2
	Pb01	RO1,RAJ2,717,133,84,189,61,206,694,397,57,351,218,MFC,JHS,PB01,7455
	1578	1578
	769	769

For the Likelihood Analysis of Geographic Range Evolution, the phylogenetic tree obtained via the program BEAST was written in parentheses (in the Newick format) and used as input information, together with the area distribution of each taxon, for analysis in the program LAGRANGE v.20110117 (distributed by the authors from http://code.google.com/p/lagrange) [36]. Three areas were settled on for the biogeographic analysis: A (corresponding to Peru, Colombia, Equador and Venezuela and also to the Brazilian states of Acre, Amazon, and Roraima), B (corresponding to the Brazilian states of Para, Rondonia, Mato-Grosso and Goias) and C (corresponding to Paraguay, Uruguay and Argentina and also to the Brazilian states of Sao Paulo, Rio de Janeiro, Parana and Minas Gerais. The maximum range size was set at 3 (areas A, B and C) while allowing 4 possible ranges (ABC, AB, BC and AC).

## Results

### Morphological analysis

The yeast cell area seems unrelated to the genetic group as no significant species variation in yeast size was found, with the only exception being Pb01, the first *P. lutzii* isolate ever reported [7], [16]; this isolate shows much larger yeast cells when compared to other isolates for the conditions herein applied. The PS2 isolates, except Pbdog and Pb927, were elongated, as reported previously [37] (Figure S1).

Eighteen out of 26 S1 isolates and the Pbdog (PS2) isolate produced conidia 2.25 to 7.99 µm wide in diameter (median value, 5.13 µm), which were more abundant in SEA than in PDA medium. Two Colombian PS3 isolates were unable to generate conidia in either culture medium. *P. lutzii* isolates produced an intermediate to high number of conidia (Table 4 and Figure S2). Most of the Pb01 conidia, and few EE conidia, both *P. lutzii* isolates, are longer (its length ranged from 2.17 to 22.89 µm, with 11.07 µm being the median value) than S1 and PS2 conidia, whose lengths ranged from 2.25 to 7.99 µm (median value, 5.13 µm). Therefore, conidial morphology appears to be an important morphological marker for differentiation between *P. lutzii* and the three cryptic species of the *P. brasiliensis* complex (S1, PS2 and PS3).

**Table 4:** Conidia production by isolates belonging to the four species of the *Paracoccidioides* genus.

Species	Isolate	Conidia production			
		PDA		SEA	
		Adhesive tape	Slide Culture	Adhesive tape	Slide Culture
**S1**	T1F1	−	−	−	−
	T3B6	−	+	++++	++++
	T4B17	−	+	++++	++++
	T5LN1	−	−	−	−
	T7F6	−	++	++++	+++
	T8LN2	−	++	++	++
	T9B1	−	++	++++	++++
	T13LN2	−	−	−	−
	T15LN1	−	++	++++	++++
	BT60	−	−	++	+
	PB18	−	−	−	−
	PB339	−	−	−	−
	S1	+	+	++	+++
	ILSL57	−	+	++++	++++
	BT85	−	−	−	+
	PB265	−	−	−	−
	D01	−	−	−	+
	D02	−	−	−	−
	D03	−	−	+++	+++
	D04	−	−	++	++
	D05	−	−	++	+
	D06	−	−	−	−
	D07	−	−	+	++++
	D09	−	−	+	+
	D10	−	−	++++	+++
	D11	−	−	++++	++
**PS2**	T10B1	−	−	−	−
	PB927	−	−	−	−
	BT84	−	−	−	−
	PB262	−	−	−	−
	PB02	−	−	+	−
	PB03	−	−	−	−
	PB04	−	−	−	−
	PBdog	+++	++	++++	++++
	1430	−	−	−	−
**PS3**	EPM83	−	−	−	−
	EPM77	−	−	−	−
***P. lutzii***	PB01	−	−	−	+
	EE	−	−	+++	+++
	Jal	−	−	−	++
	Pb8334	−	−	−	−
	Pb66	−	−	−	−

− 0.

+ up to 5.

++ from 5 to 20.

+++ from 20 to 50.

++++ >50 conidia per microscopic field.

### Single Nucleotide Polymorphism analysis

**Figure 2:**
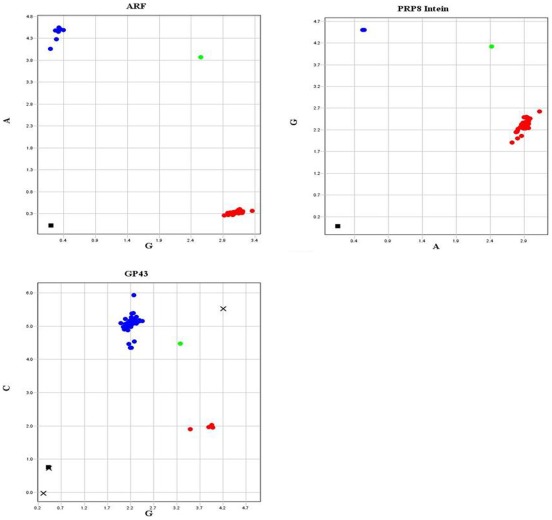
Real Time PCR with TaqMan probes for SNP detection in isolates belonging to the different species from *Paracoccidioides*genus. Endpoint fluorescence intensity graphics for: SNP3-*ARF* (G for S1, PS3 and *P. lutzii* isolates, and A for PS2 isolates), SNP4-*GP43,* exon 2 (G for S1, PS2 and *P. lutzii*, and C for PS3) and SNP5-PRP8 intein (A for S1, PS2 and PS3, and G for *P. lutzii*). Green dots: “heterozygote” additional controls, using two different DNA samples together; black squares: negative controls;“X”: undetermined samples.

Analysis of the four SNPs, two in *GP43* exon 2, and one in each of the *ARF* and PRP8 intein genes (Table 5 and Figure 2), allowed us to differentiate 34 *Paracoccidioides* isolates, not evaluated previously by MLST, into species. Of these, 30 belonged to S1 and 4 to PS2. As predicted in Table 2, the use of at least three SNPs was sufficient to individually differentiate isolates into the different cryptic species: one SNP that distinguishes the *P. lutzii* species (the SNP at position 344 of *GP43*-exon 2 or the SNP at the position 1146 of the PRP8 intein), one to differentiate PS3 isolates (the SNP at position 494 of *GP43*-exon 2) and one to distinguish the PS2 isolates (the SNP at position 298 of *ARF* gene).

**Table 5:** SNPs detected by SNaPshot and Real Time PCR (TaqMan) for different isolates from the *Paracoccidioides* genus.

Species	Isolate	SNaPshot		Real Time PCR (TaqMan)		
		SNP1 *ARF*	SNP2 Exon2-*GP43*	SNP3 *ARF*	SNP4 Exon2-*GP43*	SNP5 PRP8 intein
**S1**	T1F1*	C	T	G	NA	A
	T3B6*	C	T	NA	G	NA
	T4B17*	C	T	NA	G	NA
	T15LN1*	NA	NA	G	NA	A
	S1	C	T	G	G	A
	ILSL57	C	T	G	G	A
	Pb-baby	C	T	G	G	A
	Bt85	C	T	G	G	A
	Pb265	C	T	G	G	A
	D01	C	T	G	G	A
	D02	C	T	G	G	A
	D03	C	T	G	G	A
	D04	C	T	G	G	A
	D05	C	T	G	G	A
	D06	C	T	G	G	A
	D07	C	T	G	G	A
	D09	C	T	NA	G	NA
	D10	C	T	G	G	A
	D11	C	T	G	G	A
	EPM04*	C	T	G	G	A
	EPM46	C	T	G	G	A
	EPM48	C	T	G	G	A
	EPM85*	C	T	G	G	A
	EPM75	C	T	G	G	A
	EPM101	C	T	G	G	A
	Pb21	C	T	G	G	A
	Pb534	C	T	G	G	A
	Pb113	C	T	G	ND	A
	Pb157	C	T	G	G	A
	Pb94	C	T	G	G	A
	Pb135	C	T	G	G	A
	Pb25	C	T	G	G	A
	Pb09	C	T	NA	G	NA
	Pb728	C	T	G	G	A
	Pb698	C	T	G	G	A
	TCC	C	T	G	G	A
**PS2**	Pb927 **	T	T	A	NA	NA
	T10B1*	T	T	A	ND	A
	Bt84*	T	T	A	G	NA
	Pb262*	T	T	A	NA	NA
	Pb04*	T	T	A	NA	A
	Pbdog	T	T	A	NA	A
	1430	T	T	A	NA	A
	Pb106	T	T	A	NA	NA
	Pb22	T	T	A	NA	NA
**PS3**	EPM83*	C	T	G	C	A
	EPM77*	C	T	G	C	A
	60855*	C	T	NA	NA	NA
***P. lutzii***	Pb01*	C	G	NA	G	NA
	Pb66*	C	G	NA	G	NA
	8334*	C	G	G	G	G
	JAL *	C	G	NA	G	NA
	EE *	C	G	G	G	G

* Isolates used as positive controls.

** Isolate previously grouped in S1 species (Matute et al., 2006), but recognized as PS2 species in this study, corroborating the data found by PRP8 intein sequencing (Theodoro et al., 2008b). NA = Not Analyzed ND = Not Determined by the method.

### Inference of the biogeographic history of the genus *Paracoccidioides*

The haplotype nets constructed by using the NETWORK program are shown in Figure 1 and Table 3. As already documented by Matute et al. [14], the haplotype nets constructed using *alpha-tubulin* and *GP43* exon2 alignments are best suited for setting apart phylogenetic species [14]. On the other hand, *ARF* alignments put together S1 and PS3 isolates (B19 and C1 haplotypes). Sharing of genetic material from isolates 133 and 769 with the species S1 and PS2 is corroborated in our haplotype nets [7]. These isolates were grouped with *P. lutzii* according to a previous analysis in which hydrophobin, HSP70, ITS1-5.8S-ITS2 and KEX genes were used [7]. This genetic exchange is clear in the *alpha-tubulin* network, where the isolate 133 falls into the PS2 haplotype, because of a homoplastic character at position 172, represented by a reticulation involving the median vectors (mv) 1, 2 and 3 (Figure 1). In the *GP43* network the linkage is also clear between isolate 133 and the B15 haplotype, which encompasses PS2 isolates, and also between isolate 769 and haplotype B10, which encompasses S1 isolates.

In haplotype nets, historical demographic expansion events are characterized by star-like clusters of nodes around a founder population node. These expansion events were indicated by the Nested Clade Analysis (Table 6). In general, our results indicated a continuous expansion of S1 and a dispersal event that gave rise to PS3 in Colombia. Within S1, a possible fragmentation event gave rise to a subclade or subspecies, filled with Venezuelan isolates, as previously suggested [19].

**Table 6:** Historical events associated with *Paracoccidioides* genus biogeography, according to Nested Clade Analysis.

Locus	Historical events with statistical significance
	Within S1/PS3
*ARF*	- Simultaneous geographic expansions
	- Restricted gene flow with isolation by distance/Restricted dispersal by distance in non-sexual/asexual species (PS3).
*TUB*	- Gene flow/Restricted dispersal, but with some long distance dispersal events over intermediate areas.
	- Long distance colonization and/or fragmentation event separating Venezuelan isolates from other S1.
*GP43*	- Restricted gene flow with isolation by distance/Restricted dispersal by distance in non-sexual/asexual species (PS3).
	Between PS2 and S1/PS3
*ARF*	No historical event detected
*TUB*	No historical event detected
*GP43*	Inconclusive data
	Between *P. lutzii* and S1, PS2, PS3
*ARF*	Inconclusive data
*TUB*	No historical event detected
*GP43*	Inconclusive data

**Figure 3:**
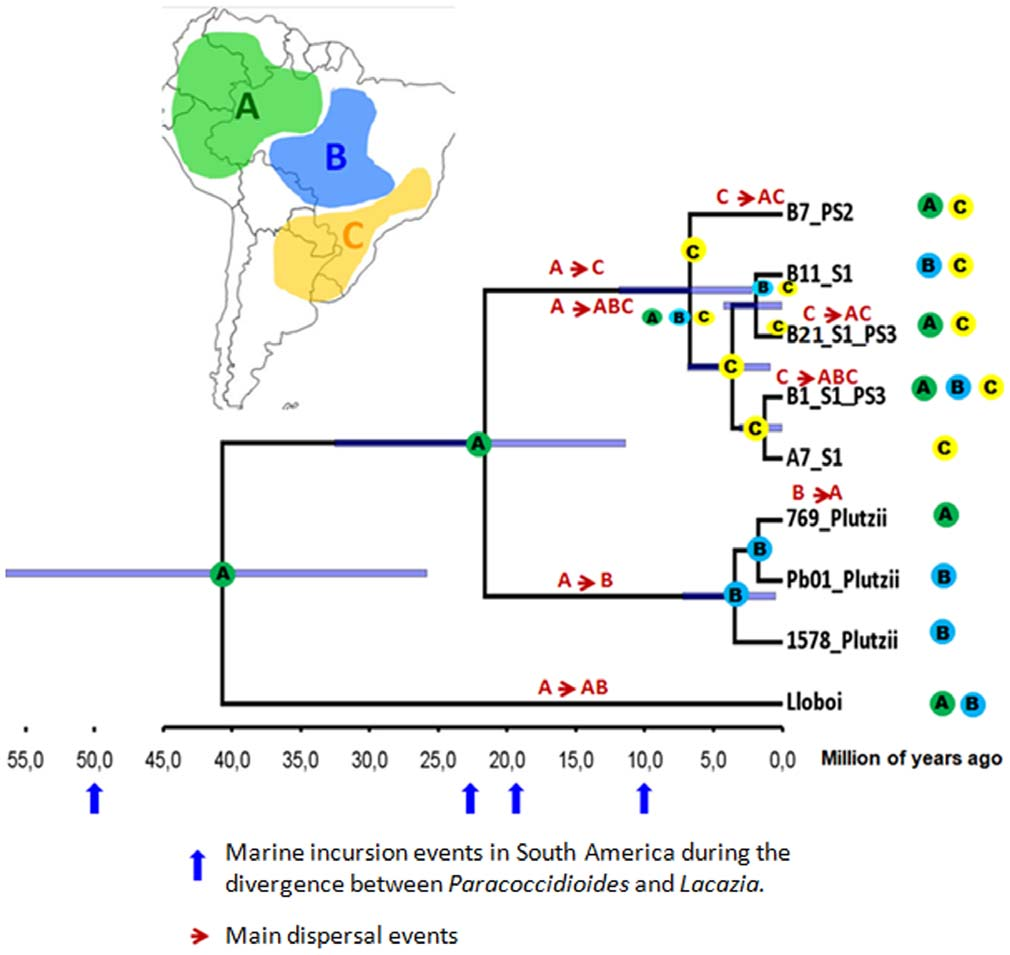
Phylogenetic tree generated with the program BEAST, ancestral areas according to Likelihood Analysis of Geographic Range Evolution. The tree was generated with the program BEAST. The blue bars illustrated the extent of the 95% highest posterior density intervals for each divergence time. The scale is in years. A, B and C are the areas settled for LAGRANGE analysis. Areas in the branch tips represent the current distribution, and areas in the nodes of the tree are the most probable ancestral areas according to LAGRANGE analysis. The main dispersal events are indicated by red arrows. Tip labels designate the name of the haplotype (same names asin Table 3, for the *ARF* gene), followed by the species found in this haplotype (S1, PS2, PS3 or *P. lutzii*).Marine incursion events, which occurred while *Lacazia* and *Paracoccidioides* genera diverged, are indicated with blue arrows along the time scale axis.

The phylogenetic tree constructed by means of BEAST indicated a divergence of around 21.6 million years (in an interval of 11–32 mya) between *P. brasiliensis* (S1, PS2 and PS3) and *P. lutzii* and 6.7 million years between S1/PS3 and PS2. In addition, according to the LAGRANGE analysis, the S1/PS3 ancestral area was “C”. Therefore, the current occurrence of some isolates (all from PS3 species) in Colombia (area A) must be due to a recent dispersal, rather than a vicariant event (Figure 3). Such events might have played an important role in the divergence among S1, PS2 and *P. lutzii*, since ancestors of both *P. lutzii* and the *P. brasiliensis* species complex share overlapped areas (A and B). The same can be suggested for the origin of PS2 and S1/PS3 isolates, whose ancestors also share an overlapped area (C). Vicariant events might have also occurred between the genus *Paracoccidioides* and its sister species *L. loboi*, because their ancestors occupied the same area (A). In Figure 3, only the node areas with the highest probability, according to LAGRANGE results, are shown.

## Discussion

In relation to the speciation of the genus *Paracoccidioides*, both a straightforward identification method and an evolutionary perspective are presented herein. The former is focused on a fast and firm molecular diagnosis of *Paracoccidioides* species, from clinical or environmental samples, suitable for epidemiological studies and eventually, for clinical use, especially if different species turn out to induce different host responses. The second view infers the possible historical events that have resulted in the current biogeography of this genus and its sister species, *L. loboi*.

### Morphological and molecular markers for *Paracoccidioides* species

Because of subtle or inexistent morphological differences among cryptic species, molecular techniques have become the tool of choice to tell them apart. For example, *Coccidioides posadasii* grows slowly in high concentrations of salt when compared to *C. immitis* [38], but this phenotype is not used for species recognition. The same is valid based on morphological studies on *Paracoccidioides*. Since a previous work [7] indicated some potential difference in yeast cell size and shape, and conidial production among the four phylogenetic species of the genus *Paracoccidioides*, a larger number of isolates, belonging to each species, were herein analyzed to better evaluate such observations and eventually establish morphological markers for species differentiation.

We observed that yeast cell size may vary in a given isolate and in isolates from the same species, but it does not distinguish among S1, PS2, PS3 and *P. lutzii*. Yet most PS2 isolates, except the Pbdog and Pb927, tend to present elongated yeast cells, similar to pseudohyphae. These isolates do not easily transform from the mycelial to the yeast phase (data not shown). In fact, one of them, the Pb22 isolate, although obtained as yeast from a human patient, is unable to grow in culture in the yeast morphology. However, to be a morphological marker these elongated yeast cells should be present in all PS2 isolates. It is important to remark that all isolates, including Pb22, were confirmed as *Paracoccidioides* isolates after ITS1-5.8S-ITS2 sequencing (data not shown).

Production of conidia, the infective asexual spore of the mycelial phase, was higher in SEA than in PDA medium, probably because the former resembles the natural conditions in which this process might occur [39]. Two thirds of S1 isolates (69%) produced conidia under these conditions; with the exception of Pbdog (PS2), all PS2 (9 samples) and PS3 isolates (2 samples) produced very few or no conidia. It is important to mention that the Pbdog isolate has other peculiarities, such as a diffusible brown pigment in agar medium and a smooth, instead of wrinkled, aspect of the yeast culture. Sequencing of the eight loci for species recognition [14] has confirmed that Pbdog does belong to the PS2 species (data not shown).

Teixeira et al. [7] observed very long conidia in Pb01, a characteristic shared with another *P. lutzii* isolate, EE. Additional studies with a larger number of *P. lutzii* isolates (Teixeira et al., in revision) have indicated that the presence of very long conidia can be considered a frequent characteristic of this species, making conidia (shape and size) potential morphological markers for differentiation between *P. lutzii* and the three cryptic species of the *P. brasiliensis* complex (S1, PS2 and PS3).

**Figure 4:**
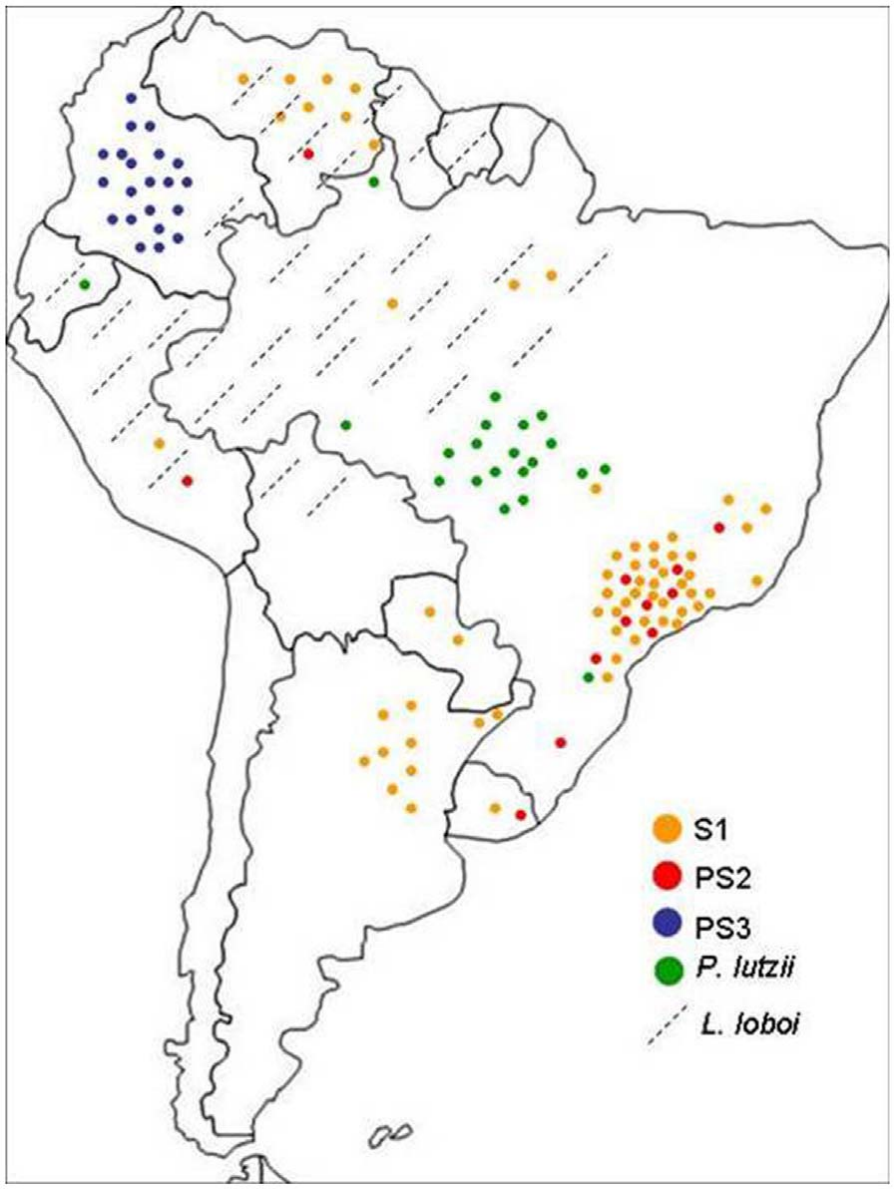
Geographic distribution of *Paracoccidioides* genus and *L. loboi.* Current distribution of *Paracoccidioides* species S1, PS2, PS3 and *P. lutzii,* and their sister species *L. loboi,* in South America (modified from [53]).

Other morphological characteristics, such as tolerance to different pHs and salt concentrations, have also been tested to address phenotypic differences among S1, PS2, PS3 and *P. lutzii* (data not shown). However, morphological studies done so far to characterize *Paracoccidioides* species have failed to do so. In fact, the observation of morphological differences among phylogenetic and very closely related species, including those from the *Paracoccidioides* genus, is a very difficult task. The only distinctive characteristic observed to date was the longer conidia in *P. lutzii* species, which, incidentally, is the most phylogenetically divergent one. On the other hand, SNP markers, such as those chosen herein, are appropriate for conclusive species recognition within the genus *Paracoccidioides*; with them it was possible to classify 34 additional isolates, which were included in a geographic distribution map of *Paracoccidioides* species in South America (Figure 4). The four SNPs were equally suitable for species differentiation because they represent fixed polymorphisms among the different species; with only 3 SNPs simultaneously applied (SNP at position 344 of *GP43*-exon 2 or SNP at position 1146 of PRP8 intein for *P. lutzii*, SNP at position 494 of *GP43*-exon2 for PS3, and SNP at position 298 of *ARF* for PS2; see Table 2), to distinguish species within the genus *Paracoccidioides*, rendering this technique a faster and simpler method than MLST, because amplification and sequencing of numerous genes for phylogenetic analysis are no longer needed. This approach has also been applied to differentiate *C. immitis* and *C. posadasii*, two cryptic species in the genus *Coccidioides* [40].

### Biogeographic history of the genus *Paracoccidioides*

Inferring the biogeographic history of *Paracoccidioides* is not a simple matter for the following reasons: i) the constant migration of human hosts; ii) the long latency period of PCM and iii) the scarcity of environmental isolates.

With the exception of one *P. lutzii* strain isolated in the southern Brazilian state of Paraná [41], there is a strong clustering in the species distribution of *Paracoccidioides*, according to their origin (Figure 4), an aspect already documented by Calcagno et al. [42] long before the discovery of cryptic species in this pathogen.

All environmental and armadillo (*D. novemcinctus; C. centralis*) isolates so far available in culture collections [43], [44]–[46], [9], [10] belong to *P. brasiliensis* S1, PS2 and PS3. They were isolated in southern Brazil (in *D. novemcinctus*) or Caldas, Colombia (in *C. centralis*). An evaluation of wild animals, mainly armadillos, in western-central Brazil, is pending in order to confirm the geographic distribution of *P. lutzii*.

NCA analysis suggests a long dispersal event, rather than a vicariant process for the emergence of PS3 species. This may have been due to migration of S1 individuals to Colombia [14], crossing preexisting geographic barriers, probably the Andean Cordillera. Likelihood Analysis of Geographic Range Evolution also corroborates a dispersal hypothesis, rather than a vicariant process, because the area “A”, in Colombia, is a derived rather than an ancestral area, since the most recent common ancestor of S1 and PS3 might have inhabited area “C”.

Since most PS3 isolates found to date come from Antioquia, Colombia [14], a region located between the Western and Central Andean Cordillera, we believe that the geographic seclusion of this location may have contributed to the isolation of PS3. Although the *ARF* phylogeny makes S1 paraphyletic, that is, it places S1 and PS3 isolates together in the same clade, the PS3 speciation by dispersal to Colombia might have occurred less than 6.7 mya, which is the estimated time for the divergence between S1/PS3 and PS2 according to the *ARF* substitution rate (Figure 3). A possible explanation for the recent speciation of PS3 could be the impact of the two major geological events that took place in South America from 60–8 mya, namely the Andes uplift and the episodic marine incursions, caused by global sea level rises during the extensional tectonic phases [47]–[49].

In contrast to the geological instability of the Andean region, the neighboring Brazilian and Guiana shields are extremely stable, not having undergone any deformation in the past 108–109 years. The Guiana shield encompasses most of the Guiana surface, southern Venezuela, southeast Colombia and northern Amazonia, while the Brazilian shield includes western-central and southeast Brazil. Between these shields lies the Amazonian depression, originated in the Paleozoic, and reactivated in the Triassic while the tectonic plates were drifting apart to render Africa and South America separate continents. After that, the convergence of tectonic plates gave rise to the Andes Cordillera, changing the drainage of the Amazonian rivers from westward to eastward. This new drainage system, and the coincident marine incursions, created a large wetland portion that allowed the interflow between fresh and marine organisms [49]. At least five marine incursions took place in South America: two independent ones in southern Parana and the northwest Amazon Basin, in the late Cretaceous (61 mya), another in the early Eocene (around 50 mya) in Colombia, a fourth in the Miocene (around 20 mya) in Colombia and in the northwestern region of the Amazon basin, and finally, also in this area, a late Miocene (11.8–7.0 mya) incursion. In this manner, a 1 million Km2 lake, denominated Pebas/Solimões, was formed [49]–[52], covering most of today's Colombian territory. So, until the late Miocene, while the genus *Paracoccidioides* was going through a radiation process in South America, the Colombian territory was submerged.

Vertebrate hosts might have played an important role in the long dispersal event that gave rise to PS3. Indeed, the long latency period, the tendency toward chronicity and the mitigation of virulence threats have been proposed as evidence for a long coexistence with vertebrates, which could have shaped the current geographic distribution of *Paracoccidioides* species. Richini-Pereira et al. [53] suggested the importance of *Xenarthra* species, particularly armadillos, in the eco-epidemiological features involving PCM and its agents. These authors demonstrated a high frequency of molecular detection of *Paracoccidioides* sp in tissue samples from different *Xenarthra* species and associated their data with the weak immune response and lower body temperature of these animals [54], [55]. In addition, the ancient origin of the *Xenarthra* order (around 65 mya, in South America) and its coexistence with fungi belonging to the family Ajellomycetaceae, suggest that these mammals could have played an important role in the evolution of parasitism in *Paracoccidioides* [5].

The remaining species from the genus *Paracoccidioides* occur almost sympatrically across their geographic range, except *P. lutzii*, which is prevalent in western-central Brazil. This regionalization suggests an alopatric speciation. However, no clear geographic barrier is known to have separated *P. lutzii* from the S1 and PS2 species. Still, the process of defining geographic barriers for microorganisms is more complicated than for more complex organisms, such as plants and animals. Small variations in weather, temperature, humidity and environmental resources might create ecological barriers sufficient to support species divergence and emergence of adaptations. Since the geographic distance between *P. lutzii* and S1/PS2 is relatively short, their divergence may have resulted from a reproductive isolation leading to a parapatric speciation due to ecological variation, rather than distance, as observed for PS3. A restricted gene flow between these two populations could have been intensified by natural selection and genetic drift. In order to better explore this hypothesis, the interaction of these pathogens with saprobic substrates should be evaluated. Until now, this point has been addressed in a single study [39], in which fungal growth and conidial production in different soil textures and different water availabilities were analyzed and revealed that S1 isolates produced many more conidia than PS2.

According to Teixeira et al. [7], two *P. lutzii* isolates (769 and 133) share alleles with S1 and PS2 *P. brasiliensis*, respectively. As proposed by these authors, these isolates could be either hybrids or possessors of ancestral polymorphisms in their genomes. The absence of geographic barriers could contribute to interspecific matching and mating between different *Paracoccidioides* species, a possibility not to be discarded since genomic and morphological data revealed genes related to a sexual cycle in this genus [56].

Usually, the sympatric speciation in fungal pathogens is related to a specialization process towards different hosts, as happened in *Pneumocystis* [57] and *Botrytis* [58] species, for example. However, this possibility does not appear applicable for explaining S1 and PS2 sympatry, given that they infect the same hosts, human and armadillos. The probability of substrate and resource preferences in their saprobic lifestyles (disruptive selection) may be considered a manner of establishing a different adaptation processes in nature [59]. In addition, according to the neutral theory, sympatric speciation may also occur, even without geographic and ecological barriers, ecological gradients or natural selection [60].

Although Matute et al. [7] have considered S1 a single species despite its paraphyletic nature, we are led to suppose that a partial and insufficient sampling may be masking the true phylogeography of S1 isolates on account of its wide geographic distribution together with the fragmentation observed in the formation of a Venezuelan subpopulation [19]. We believe that ecological studies, such as those that aim to isolate or detect the cryptic species in the environment, rather than in migratory patients, would be helpful to clarify some uncertainties about the speciation within the genus *Paracoccidioides*.

### Divergence between the genus *Paracoccidioides* and its sister species, *L. loboi*

Although *L. loboi* belongs to the Ajellomycetaceae family [6], [7], as a sister species to the genus *Paracoccidioides*, it displays a peculiar lifestyle when compared to other Ajellomycetacean pathogens: i) it has been impossible to culture in vitro, until now; ii) besides humans, it infects dolphins, and is hence associated with aquatic environments, and iii) it causes a subcutaneous, not systemic, mycosis both in humans and dolphins [8]. The deep whole genomic comparisons between *L. loboi* and *Paracoccidioides* would be extremely helpful to understand the different niches occupied by these two genera. Expansions and contractions in gene families and genome reduction are probably the reason for the specialized niche of *L. loboi*.

Lacaziosis is prevalent in tropical and subtropical areas, particularly in the Amazon Basin (Brazil and Colombia) (Figure 3), and also has been reported in Costa Rica, Venezuela, Peru, French Guiana, Suriname, Panama, Guiana, Ecuador, Bolivia, Mexico, Canada and United States [61]. A single European case was described in a dolphin caretaker [62] and the first reported case in the US came from a patient who had traveled to Venezuela [63].

Two dolphin species, *Tursiops truncatus* (marine) and *Sotalia fluviatilis* (fluvial), are responsible for the current distribution of *L. loboi*. The first species has a global distribution in tropical and equatorial regions while the second occurs in the Amazon Basin, from the Atlantic coast of Florida in the United States to Paraná state (Brazil), and in the Suriname rivers [64]–[66].

Since many cases of lacaziosis are the result of transmission from dolphins to humans, water is believed to be the environmental source of the pathogen as well as the reason for its large geographic distribution [61]. Bagagli et al. [67] put forward a hypothesis according to which *L. loboi* may be in an evolutionary phase of strong reduction of the saprobic form and specialization towards parasitism, so that it depends on the host for its growth and dissemination. If this hypothesis is confirmed, the large geographic distribution of this pathogen will be associated with host migration rather than water itself. The high prevalence of *L. loboi* in the Amazon Basin (Figure 4) and its phylogenetic proximity to all members of the genera *Paracoccidioides* and *Histoplasma*, suggest that this peculiar Ajellomycetacean has emerged in South America, as have the other two.

According to Figure 3, the ancestral population of *Paracoccidioides* and *L. loboi* lived in the northern region of South America (area A), around 40.6 mya. While being phylogenetically close to *Paracoccidioides*, *L. loboi* inhabits a different ecological niche (aquatic vs. soil), and causes a subcutaneous (not systemic) mycosis, transmitted by trauma and not by inhalation of spores. The habitat of this pathogen, mainly in the Amazon Basin, corroborates the vicariant event suggested by our LAGRANGE analysis, and explains the divergence of the ancestral population that gave rise to *Lacazia* and *Paracoccidioides*. This event may have been triggered by the formation of new and empty ecological niches (wetland and/or totally submerged regions), and the simultaneous emergence of riverine Cetacean mammals in the Oligocene and early Miocene [68], [69], in which *L. loboi* ancestors may have found an ideal refuge away from the continuous instability represented by those lacustrine environments.

## Supporting Information

Figure S1 **Yeast morphology.** Aspect of yeast cells from isolates belonging to *P. lutzii* (A), PS2 (B), PS3 (C) and S1 (D and D′) species.
(TIF)

Figure S2 **Conidial morphology.** Production of conidia and their morphological aspects in isolates belonging to S1 (A and A′), PS2 (B) and *P. lutzii* species (C).
(TIF)

Table S1 **Primers used to amplify the SNP-containing genes evaluated by the SNaPshot technique.**
(DOC)

Table S2 ***Paracoccidioides*** **isolates used in Nested Clade Analysis and their respective geographic areas.**
(DOC)
