# Supplementary figures and images for: Genus Paracoccidioides: Species Recognition and Biogeographic Aspects
Source: PLoS One. 2012 May 30;7(5):e37694. doi: 10.1371/journal.pone.0037694 (PMC3364295; doi:10.1371/journal.pone.0037694)

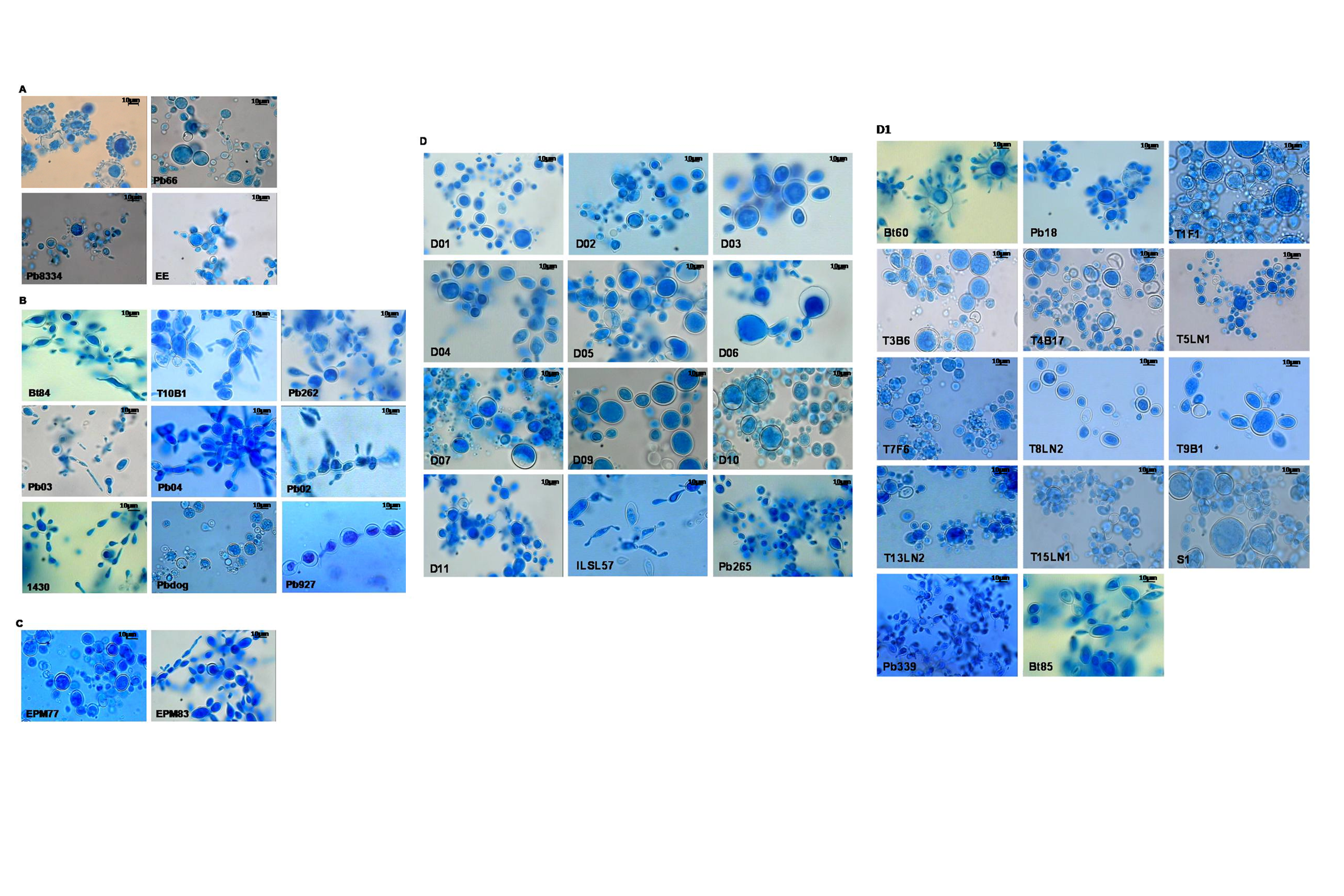

Supplement: Figure S1 — Yeast morphology. Aspect of yeast cells from isolates belonging to P. lutzii (A), PS2 (B), PS3 (C) and S1 (D and D′) species. (TIF) [file pone.0037694.s001.tif]

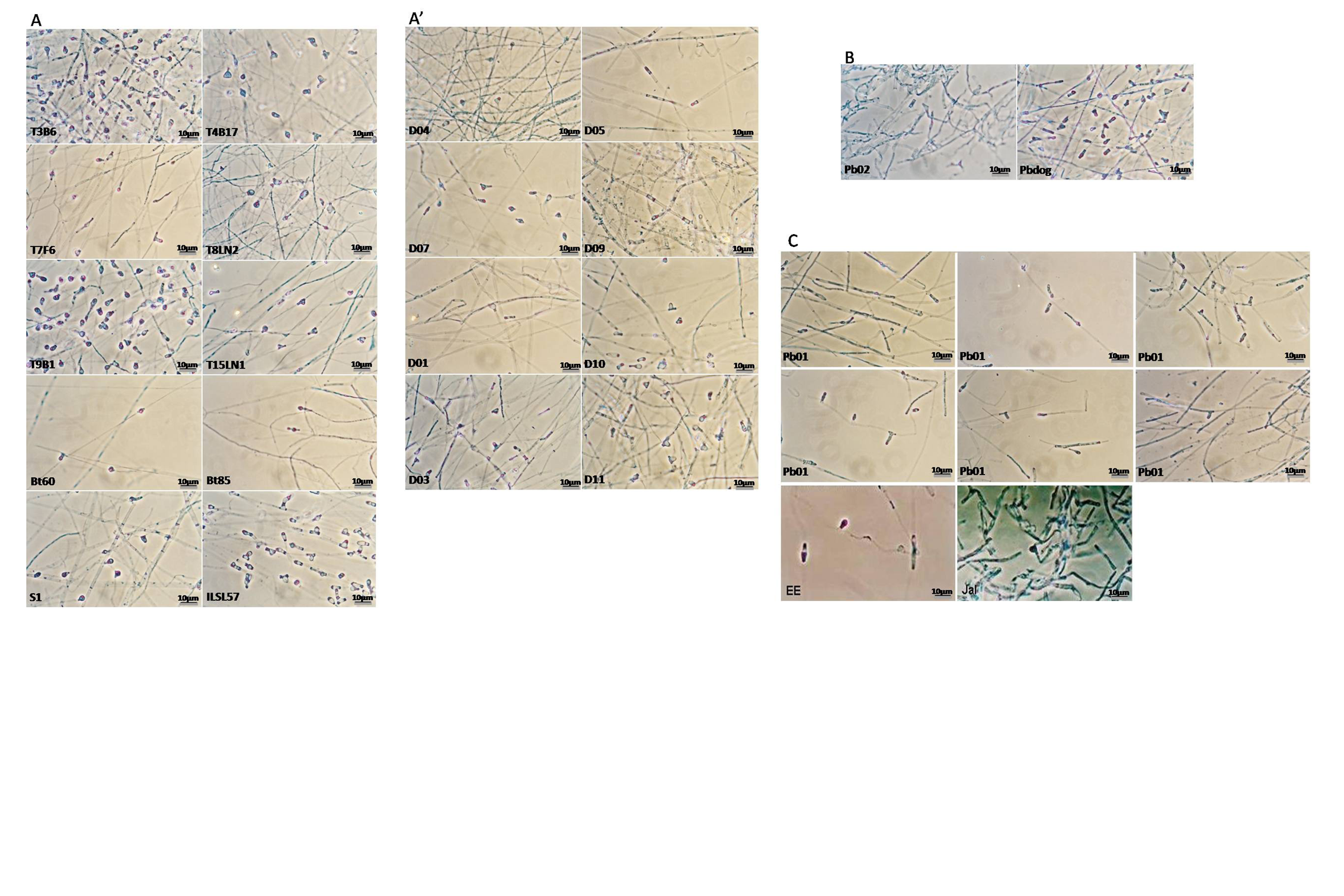

Supplement: Figure S2 — Conidial morphology. Production of conidia and their morphological aspects in isolates belonging to S1 (A and A′), PS2 (B) and P. lutzii species (C). (TIF) [file pone.0037694.s002.tif]
