# Supplementary material for: Genus Paracoccidioides: Species Recognition and Biogeographic Aspects
Source: PLoS One. 2012 May 30;7(5):e37694. doi: 10.1371/journal.pone.0037694 (PMC3364295; doi:10.1371/journal.pone.0037694)
Supplement: Table S1 — Primers used to amplify the SNP-containing genes evaluated by the SNaPshot technique. (DOC) [file pone.0037694.s003.doc]

**Supporting Information**

**S1**: Primers used to amplify the genes containing the SNPs evaluated by the SNaPshot technique.

| **Gene** | ***Primer*** | **Sequence 5’-3’** | **Amplicon** | **Reference** |
| --- | --- | --- | --- | --- |
| *GP43* | *GP43*-E2-fwd | CTAGAATATCTCACTCCCAG | 723pb | This work |
|  | *GP43*-E2-rev | GCCCCCTCCGTCTTCCATGTCC |  | Matute et al., 2006 |
| *ARF* | *ARF* – fwd | tctcatggttggcctcgatgctgcc | 407pb | Matute et al., 2006 |
|  | *ARF*-rev | gagcctcgacgacacggtcacgatc |  |  |
