# Supplementary material for: Genus Paracoccidioides: Species Recognition and Biogeographic Aspects
Source: PLoS One. 2012 May 30;7(5):e37694. doi: 10.1371/journal.pone.0037694 (PMC3364295; doi:10.1371/journal.pone.0037694)
Supplement: Table S2 — Paracoccidioides isolates used in Nested Clade Analysis and their respective geographic areas. (DOC) [file pone.0037694.s004.doc]

**Table S2:** *Paracoccidioides* isolates used in Nested Clade Analysis and their respective geographic areas.

| **Isolate (species)** | **Reference** | **Area** | **Latitude***** | **Longitude***** |
| --- | --- | --- | --- | --- |
| **B1 (S1)** | Matute et al. (2006a) | São Paulo/ Brazil | -23.55 | -46.64 |
| **B2 (S1)** | Matute et al. (2006a) |
| **B4 (S1)** | Matute et al. (2006a) |
| **B3 (S1)** | Matute et al. (2006a) |
| **B5 (S1)** | Matute et al. (2006a) |
| **B6 (S1)** | Matute et al. (2006a) |
| **B7 (PS2)** | Matute et al. (2006a) |
| **B8 (S1)** | Matute et al. (2006a) |
| **B9 (S1)** | Matute et al. (2006a) |
| **B10 (S1)** | Matute et al. (2006a) |
| **B14 (S1)** | Matute et al. (2006a) |
| **B15 (PS2)** | Matute et al. (2006a) |
| **B17 (S1)** | Matute et al. (2006a) |
| **B20 (S1)** | Matute et al. (2006a) |
| **B22 (S1)** | Matute et al. (2006a) |
| **B23 (PS2)** | Matute et al. (2006a) |
| **B25 (S1)** | Matute et al. (2006a) |
| **B26 (PS2)** | Matute et al. (2006a) |
| **C1 (PS3)** | Matute et al. (2006a) | Colômbia | 4.60 | -74.08 |
| **C2 (PS3)** | Matute et al. (2006a) |
| **C3 (PS3)** | Matute et al. (2006a) |
| **C4 (PS3)** | Matute et al. (2006a) |
| **C5 (PS3)** | Matute et al. (2006a) |
| **C6 (PS3)** | Matute et al. (2006a) |
| **C7 (PS3)** | Matute et al. (2006a) |
| **C8 (PS3)** | Matute et al. (2006a) |
| **C9 (PS3)** | Matute et al. (2006a) |
| **C10 (PS3)** | Matute et al. (2006a) |
| **C11 (PS3)** | Matute et al. (2006a) |
| **C12 (PS3)** | Matute et al. (2006a) |
| **C13 (PS3)** | Matute et al. (2006a) |
| **C14 (PS3)** | Matute et al. (2006a) |
| **C15 (PS3)** | Matute et al. (2006a) |
| **C16 (PS3)** | Matute et al. (2006a) |
| **C17 (PS3)** | Matute et al. (2006a) |
| **C18 (PS3)** | Matute et al. (2006a) |
| **C19 (PS3)** | Matute et al. (2006a) |
| **C21 (PS3)** | Matute et al. (2006a) |
| **C20 (PS3)** | Matute et al. (2006a) |
| **V1 (S1)** | Matute et al. (2006a) | Venezuela | 10.48 | -66.91 |
| **V2 (PS2)** | Matute et al. (2006a) |
| **V3 (S1)** | Matute et al. (2006a) |
| **V4 (S1)** | Matute et al. (2006a) |
| **V5 (S1)** | Matute et al. (2006a) |
| **V6 (S1)** | Matute et al. (2006a) |
| **A1 (S1)** | Matute et al. (2006a) | Argentina | -27.42 | -58.98 |
| **A2 (S1)** | Matute et al. (2006a) |
| **A4 (S1)** | Matute et al. (2006a) |
| **A5 (S1)** | Matute et al. (2006a) |
| **A6 (S1)** | Matute et al. (2006a) |
| **A7 (S1)** | Matute et al. (2006a) |
| **A8 (S1)** | Matute et al. (2006a) |
| **A3 (S1)** | Matute et al. (2006a) |
| **B21 (S1)** | Matute et al. (2006a) | Paraná/ Brazil | -25.43 | -49.23 |
| **B24 (S1)** | Matute et al. (2006a) |
| **PBDOG (PS2)** | Bosco et al. (not published) |
| **B12 (S1)** | Matute et al. (2006a) | Minas Gerais/ Brazil | -19.82 | -43.96 |
| **B13 (PS2)** | Matute et al. (2006a) |
| **P2 (S1)** | Matute et al. (2006a) | Paraguay | -25.41 | -57.12 |
| **P1 (S1)** | Matute et al. (2006a) |
| **B11 (S1)** | Matute et al. (2006a) | Pará/ Brazil | -1.46 | -48.50 |
| **B16 (S1)** | Matute et al. (2006a) | Rio de Janeiro/ Brazil | -22.90 | -43.21 |
| **PE1 (S1)** | Matute et al. (2006a) | Peru | -8.35 | -74.54 |
| **U1 (PS2)** | Matute et al. (2006a) | Uruguay | -34.94 | -55.51 |
| **7455 (*P. lutzii)*** | Matute et al. (2006a) | Ecuador | -2.21 | -79.93 |
| **RO1** *(P. lutzii)*** | Teixeira et al. (not published) | Rondônia/ Brazil | -11.44 | -61.45 |
| **769 (*P. lutzii*)** | Teixeira et al. (2009) | Roraima/ Brazil | 2.74 | -60.58 |
| **Raj2 (*P. lutzii*)** | Teixeira et al. (2009) | Mato-Grosso/ Brazil | -15.60 | -56.10 |
| **717 (*P. lutzii*)** | Teixeira et al. (2009) |
| **133 (*P. lutzii*)** | Teixeira et al. (2009) |
| **84 (*P. lutzii*)** | Teixeira et al. (2009) |
| **189 (*P. lutzii*)** | Teixeira et al. (2009) |
| **61* (*P. lutzii*)** | Teixeira et al. (not published) |
| **206 (*P. lutzii*)** | Teixeira et al. (2009) |
| **694* (*P. lutzii*)** | Teixeira et al. (2009) |
| **397 (*P. lutzii*)** | Teixeira et al. (2009) |
| **57 (*P. lutzii*)** | Teixeira et al. (2009) |
| **351* (*P. lutzii*)** | Teixeira et al. (2009) |
| **218 (*P. lutzii*)** | Teixeira et al. (2009) |
| **MF** (*P. lutzii*)** | Teixeira et al. (not published) |
| **JHS** (*P. lutzii*)** | Teixeira et al. (not published) |
| **1578** (*P. lutzii*)** | Teixeira et al. (not published) | Goiás/ Brazil | -16.68 | -49.25 |
| **Pb01 (*P. lutzii*)** | Teixeira et al. (2009) |
| **B19 (S1)** | Matute et al. (2006a) |

* Isolates not evaluated for *GP43* gene. ** Isolates not evaluated for both *GP43* and *TUB* genes. ***Geographic coordinates of the main cities for PCM occurrence.
